# Neuroscience Exposure as a Predictor of Teaching Self-Efficacy

**DOI:** 10.3390/ejihpe15050086

**Published:** 2025-05-16

**Authors:** Ana Julia Ribeiro, Rafael Lima Dalle Mulle, Fernando Eduardo Padovan-Neto

**Affiliations:** 1 Laboratory of Neuropsychopharmacology of Neurodegenerative Diseases, Department of Psychology, Faculty of Philosophy, Sciences and Letters of Ribeirão Preto, University of São Paulo, São Paulo 05508-220, Brazil; anajulia.ribeiro@usp.br; 2 Laboratory of Research and Integration in Psychology, Education and Technology, Department of Psychology, Faculty of Philosophy, Sciences and Letters of Ribeirão Preto, University of São Paulo, São Paulo 05508-220, Brazil; rafael.mulle@alumni.usp.br

**Keywords:** neurosciences, self-efficacy, teachers, educational neuroscience, basic education

## Abstract

Teaching self-efficacy refers to a teacher’s confidence in their ability to engage students and foster learning, directly influencing their instructional planning, strategies, and student assessment practices. Neuroscience education for teachers has been shown to increase enthusiasm and support professional growth by introducing essential brain-related principles. This study investigated whether prior exposure to neuroscience predicts teaching self-efficacy among Brazilian basic education teachers. A total of 1120 teachers completed online surveys, providing sociodemographic information, educational background, teaching experience, and data regarding their previous neuroscience exposure. Participants’ neuroscience knowledge was assessed through a questionnaire designed to measure familiarity with fundamental neuroscience concepts, and teaching self-efficacy was evaluated using the Teacher Sense of Efficacy Scale (TSES). The results indicated that teachers with prior exposure to extracurricular neuroscience courses demonstrated significantly higher neuroscience knowledge. Additionally, those with previous neuroscience exposure exhibited a marginally significant increase in self-efficacy for instructional strategies and a significant increase in classroom management, while no significant differences were observed in student engagement. Regression analyses confirmed that neuroscience exposure significantly predicted self-efficacy in instructional strategies and classroom management. These findings reinforce the connection between neuroscience education and enhanced teaching self-efficacy, underscoring the importance of neuroeducation programs as valuable tools for supporting teachers’ professional development and well-being.

## 1. Introduction

The educational field has evolved significantly over time, with transformations occurring in response to societal needs and emerging research. These changes primarily relate to the methods of mediation between knowledge and the individuals involved in this educational process. As teaching methodologies have developed, they have created opportunities for more systematic investigation into effective learning practices. This evolution of educational approaches has opened up a rich field of research that involves identifying key variables, exploring relevant themes, and examining their complex relationships. From this perspective, the knowledge from neuroscience has become relevant, offering relevant insights into how the brain processes information, regulates emotions, and supports learning (Gkintoni et al., 2023a; Wang et al., 2023).

Within this expanding research landscape, neuroscience has emerged as a particularly promising domain, gaining prominence by offering insights into the fundamental aspects of teaching and learning processes. Neuroscientific research is increasingly focused on identifying the most effective practices and strategies for optimizing educational outcomes (Gkintoni et al., 2023b). The application of neuroscience to education extends beyond cognitive processes alone; consequently, neurosciences can also provide important perspectives on psychological factors that influence learning, including students’ motivation, beliefs in academic success, goal-setting behaviors, and academic persistence (Blackwell et al., 2007; Schroder et al., 2014; Yeager & Dweck, 2012; Yeager et al., 2019).

Another key construct of interest is self-efficacy, defined as an individual’s belief in their own ability to achieve specific goals and complete tasks successfully (Bandura, 1997, 2018). According to Bandura (2001), self-efficacy has four main sources: (a) direct experience—based on one’s own experiences; (b) vicarious experience—the ability to learn from others’ experiences; (c) social persuasion—when the social environment fosters the perception that an individual has the necessary abilities; and (d) emotional states—which can influence how individuals perceive their ability and competence to address a specific situation.

In education, self-efficacy has been widely studied across various contexts and populations (Artino, 2012; Jederlund & von Rosen, 2022; MacNabb et al., 2006). Specifically, teachers’ self-efficacy refers to their perception of their capabilities to achieve desired outcomes in student engagement and learning (Tschannen-Moran & Hoy, 2001). This construct profoundly influences teachers’ professional identity, instructional approaches, and classroom practices (Casanova & Azzi, 2015). Research demonstrates that teacher self-efficacy enhances teaching quality through improved cognitive activation, classroom management, and student support (Burić et al., 2024). Teachers with higher self-efficacy not only receive better teaching evaluations (Klassen & Tze, 2014) but also implement more effective classroom strategies (Poulou et al., 2019), ultimately leading to enhanced student outcomes, including improved academic achievement and engagement (Perera & John, 2020). Recognizing these benefits, professional development programs that strengthen teacher self-efficacy represent a valuable pathway to improving both teaching effectiveness and student performance (Perera et al., 2019).

Several studies have examined how neuroscience training influences teachers’ self-efficacy. For instance, a neuroscience program in Liberia enhanced teachers’ self-efficacy, sense of responsibility for student outcomes, and teaching motivation (Brick et al., 2021). In the United States, neuroscience programs designed for middle school science teachers reported increased enthusiasm about teaching among participants (MacNabb et al., 2006). Programs such as the BrainU workshops (Dubinsky et al., 2019) demonstrate that essential neuroscience concepts can be effectively conveyed to educators, positively influencing their professional development and ultimately affecting students’ perceptions of their learning (Dubinsky et al., 2013; MacNabb et al., 2006). Similar investigations have been conducted in Brazil. Lima et al. (2020) explored primary school teachers’ perceptions and knowledge of neuroscience before and after a continuing education course called “Neuroscience’s Course Applied to Education”. Twenty-eight teachers participated, and they unanimously deemed the course highly relevant for acquiring new neuroscience knowledge, agreeing that the concepts learned could improve their teaching practices and environments. Thus, exposure to neuroscience principles appears to positively influence teachers’ perceptions of their performance, capabilities, and professional confidence.

For the purposes of this research, we define ‘neuroscience exposure’ as the extent to which an individual has engaged with neuroscience content through formal or informal educational channels, including participation in extracurricular neuroscience courses, such as workshops, continuing education programs, or online courses focused on general neuroscience or brain knowledge.

### Current Study

The literature highlights the role of neuroscience in shaping teachers’ self-efficacy, raising questions about how these two domains intersect. A recent study by our group (Ribeiro et al., 2025) investigated whether prior neuroscience exposure (categorized as no exposure, extracurricular neuroscience courses, neuroscience-related classes, or both) predicts self-efficacy among undergraduate students. The findings revealed that students with greater exposure to neuroscience (i.e., have taken both neuroscience-related extracurricular courses and classes) demonstrated higher self-efficacy. Thus, this study underscores the potential of neuroscience-related content to improve an individual’s confidence in achieving goals, contingent on the relevant context. These results highlight a need for further investigation into whether prior exposure to neuroscience can similarly predict self-efficacy in other populations. If such an association holds among undergraduates regarding their perceived competencies in higher education, our current study questions whether the same relationship exists among teachers. Therefore, we aim to determine whether prior neuroscience exposure can serve as a predictor of teaching self-efficacy in Brazilian basic education teachers. In alignment with this objective, our study addresses the following research questions:

1. Does neuroscience knowledge differ among Brazilian basic education teachers based on the institution type, educational level, length of teaching experience, and previous neuroscience exposure?
2. Does teaching self-efficacy vary across institution types, educational levels, length of teaching experience, and previous neuroscience exposure in Brazilian basic education teachers?
3. What are the significant predictors of higher teaching self-efficacy among Brazilian basic education teachers, considering sociodemographic factors, teaching profiles, and previous exposure to neuroscience?

In this regard, we hypothesized that teachers with prior exposure to neuroscience training (i.e., extracurricular neuroscience courses) show higher levels of teaching self-efficacy compared to those without prior training, even after accounting for sociodemographic factors and teaching profiles.

## 2. Materials and Methods

### 2.1. Study Design and Participants

Data for this observational, cross-sectional study were collected via an online self-report survey (Google Forms) from November 2022 to January 2023. Participants were recruited using a snowball sampling technique through digital media platforms and email invitations. The inclusion criteria were as follows: aged between 18 and 59 years, employment as a Brazilian middle or high school teacher, possession of a bachelor’s degree, voluntary consent to participate without compensation, and complete responses to all survey items. A total of 1120 participants met the inclusion criteria. This study: (1) assessed participants’ prior exposure to extracurricular neuroscience courses; (2) evaluated their general neuroscience knowledge using a multiple-choice (true/false) questionnaire; (3) measured teaching self-efficacy using the short version of the Teacher Self-Efficacy Scale (TSES); and (4) identified sociodemographic and neuroscience-related predictors associated with self-efficacy. The study design followed a similar approach to Ribeiro et al. (2025) in analyzing these variables within this target population.

### 2.2. Ethics

The study protocol received approval from the Research Ethics Committee of the Faculty of Philosophy, Sciences, and Letters of Ribeirão Preto, University of São Paulo (FFCLRP/USP; CAAE 47436621.3.0000.5407). Participants’ personal data were managed in accordance with the guidelines of Brazil’s General Data Protection Law (LGPD, Law No. 13.709/2018). Involvement in the study was entirely voluntary, and electronic informed consent was secured via an online form. Participants were duly informed about the study’s objectives, potential risks, and their rights, and were provided with the researchers’ contact details for any further inquiries.

### 2.3. Survey Development

A self-report questionnaire was developed to collect sociodemographic data, including age, gender, ethnicity, and teaching profile information such as educational level (bachelor’s degree, postgraduate certificate, or master’s/doctoral degree). In this study, a bachelor’s degree indicated a basic undergraduate qualification, a postgraduate certificate represented specialized training beyond a bachelor’s, and master’s/doctoral degrees indicated advanced academic and research training. Additional information collected included the length of teaching experience, institution type (public, private, or both), grade levels taught (middle school, high school, or both), and teachers’ fields of knowledge. The fields of knowledge were grouped according to the São Paulo Research Foundation classification (FAPESP, 2018) based on participants’ bachelor’s degrees, and further categorized into five broad areas to facilitate analysis: (1) Natural Sciences (Biological Sciences, Health Sciences, Exact and Earth Sciences, and Agricultural Sciences), (2) Engineering and Technology, (3) Social Sciences (Applied Social Sciences and Humanities), (4) Arts and Humanities (Linguistics, Literature, and Arts), and (5) Interdisciplinary (fields not fitting other categories).

After completing the sociodemographic questions, the participants responded to the neuroscience exposure questionnaire (i.e., extracurricular neuroscience courses taken, and perceived relevance of neuroscience knowledge), the general neuroscience knowledge questionnaire, and the TSES. All survey materials were administered in Brazilian Portuguese. A comprehensive description of these instruments is provided below.

### 2.4. Measures

#### 2.4.1. Neuroscience Exposure

Prior exposure to neuroscience training was quantified by the participants’ reported attendance at extracurricular neuroscience courses focused on general neuroscience or brain knowledge (i.e., workshops, continuing education programs, or online courses), categorized as no exposure, 1–3 courses, or 4 or more courses. This categorization allows for a nuanced examination of how different types and intensities of neuroscience exposure might influence teaching self-efficacy.

Additionally, as complementary data to assess the participants perceptions on the relevance of neuroscience, they rated their agreement on a 5-point Likert scale (1 = “strongly disagree” to 5 = “strongly agree”) with the following statements: (1) the dialogue between educators and neuroscientists is important, and (2) scientific knowledge about the brain and its influence on learning is valuable for my teaching practice. In addition, the participants reported on a 5-point Likert scale (1 = “never” to 5 = “very frequently”) how often they (1) read specialized scientific journals, (2) read popular science magazines, (3) received brain-related information at their workplace (e.g., lectures, workshops), and (4) sought scientific information on the internet (Dekker et al., 2012; Macdonald et al., 2017).

#### 2.4.2. General Neuroscience Knowledge

General neuroscience knowledge was assessed through a questionnaire consisting of 17 true/false statements about the brain, drawn from prior research studies (Dekker et al., 2012; Macdonald et al., 2017). This instrument, previously translated to Portuguese and adapted for Brazilian populations (Gonchoroski, 2014; Herculano-Houzel, 2002; Ribeiro et al., 2025; Simões et al., 2022), is not a psychometric scale but a general knowledge questionnaire; therefore, it does not require validation procedures such as reliability analyses.

#### 2.4.3. Teachers’ Self-Efficacy Survey

Teachers’ self-efficacy was evaluated using the short version of the Teacher Self-Efficacy Scale (TSES) (Tschannen-Moran & Hoy, 2001), previously adapted for the Brazilian context (Casanova & Azzi, 2015). This scale consists of 12 items divided into three dimensions (4 items each) as follows: (1) efficacy in student engagement, reflecting teachers’ perceptions of their ability to motivate and engage students; (2) efficacy in instructional strategies, reflecting confidence in selecting, adapting, and implementing teaching and evaluation strategies; and (3) efficacy in classroom management, addressing teachers’ confidence in managing classroom behavior and effectively supervising students. The TSES uses a 10-point rating scale (0 = “nothing” to 10 = “a great deal”) and has demonstrated high validity and reliability (Casanova & Azzi, 2015).

### 2.5. Variable Selection

Variables for inferential analysis were selected based on their relevance to understanding teaching self-efficacy in Brazilian basic education. Sociodemographic factors, including gender and age, were selected due to their established impact on teachers’ experiences and self-efficacy (Abdel-Hadi, 2020; Sak, 2015). Teaching profile variables, including educational level, length of teaching experience, institution type, and prior exposure to neuroscience, were selected based on previously established or hypothesized associations with teaching performance outcomes (Klassen & Tze, 2014; MacNabb et al., 2006; Specht et al., 2015).

Furthermore, in Brazil, public and private basic education institutions differ considerably in resources, infrastructure, and academic performance. Private schools often offer better facilities and greater operational efficiency, frequently leading to higher student achievement. Conversely, public schools face resource limitations and administrative challenges despite their foundational principles of equity and universal education (Ferreira, 2017; Sampaio & Guimarães, 2009).

Thus, these variables were selected to capture specific characteristics of the Brazilian educational system, allowing for meaningful comparisons and insights into how neuroscience knowledge and self-efficacy relate to teaching and demographic factors across diverse educational contexts.

### 2.6. Data Analysis

Analyses were performed using Just Another Statistics Program (JASP 0.19.1.0). The Shapiro–Wilk test was applied to assess the normality of the data. The internal consistency reliability of the TSES was determined using Cronbach’s alpha (α). Prior to conducting analyses of variance (ANOVA), the homogeneity of variances was examined using Levene’s test. A significance level of *p* < 0.05 was adopted for all statistical tests.

Descriptive statistics summarized the sociodemographic characteristics, teaching profiles, previous neuroscience exposure, general neuroscience knowledge, and TSES results. One-way ANOVA compared the TSES scores and general neuroscience knowledge performance across educational levels, length of teaching experience, institution types, and neuroscience exposure levels (no exposure, 1–3 courses, and 4 or more extracurricular neuroscience courses). Eta squared (*η*^2^) was used to calculate the effect sizes, with 0.01 considered small, 0.06 medium, and 0.14 large (Cohen, 1988). Post hoc Tukey tests identified specific group differences. Cohen’s d assessed the post hoc effect sizes, with 0.20 considered small, 0.50 medium, and 0.80 large effects (Cohen, 1988).

Three separate multiple linear regression analyses were conducted, one for each dimension of the TSES (instructional strategies, classroom management, and student engagement), to identify the predictive effects of neuroscience exposure, length of teaching experience, educational level, gender, and age on teacher self-efficacy. Linear model assumptions were assessed using residual and Q-Q plots. The results are presented as mean ± SEM. Statistical significance was defined as *p* < 0.05 unless otherwise indicated. For some multiple comparisons, statistical values are reported as ranges. The levels of significance are indicated as follows: * *p* < 0.05, ** *p* < 0.01, *** *p* < 0.001, and **** *p* < 0.0001.

The combination of descriptive statistics, ANOVA, post hoc tests, and multiple linear regressions was chosen to comprehensively address the study’s objective of examining whether neuroscience exposure predicts teaching self-efficacy. Descriptive analyses allowed for an initial characterization of the sample, while ANOVA and post hoc comparisons enabled the identification of differences across groups based on the educational and professional variables. The use of multiple linear regression was essential to assess the unique predictive value of neuroscience exposure on different dimensions of teaching self-efficacy while controlling for potential confounders. This multifaceted analytical approach ensured statistical rigor and provided a deeper understanding of the relationships between prior neuroscience exposure and teacher self-efficacy beliefs.

## 3. Results

### 3.1. Sociodemographic and Teaching Profile

The study sample included 1120 participants, with a majority being women (72.41%; *n* = 811). The average age of all participants was 40.99 years (SD = 8.89), with the age range spanning from 21 to 59 years. The mean age for women was 41.08 years (SD = 8.70), and for men, 40.77 years (SD = 9.39). Regarding self-identified ethnicity, most participants identified as White (57.95%; *n* = 649), followed by Brown (27.95%; *n* = 313). In terms of region of residence, the majority lived in the Southeast region (75.71%; *n* = 848).

Regarding the teaching profile, most participants held a postgraduate certificate (41.88%; *n* = 469), followed by those with a bachelor’s degree (38.04%; *n* = 426). Teaching experience was predominantly between 10 and 20 years (31.52%; *n* = 353). Most participants worked in public institutions (56.16%; *n* = 629) and taught at both middle and high school levels (54.46%; *n* = 610). As for the fields of knowledge, the participants mainly taught in the Social Sciences (37.41%; *n* = 419) and Natural Sciences (36.79%; *n* = 412). Table A1 (Appendix A) displays additional sociodemographic characteristics and teaching profile details of the participants.

### 3.2. Previous Neuroscience Exposure and General Knowledge

**Figure 1:**
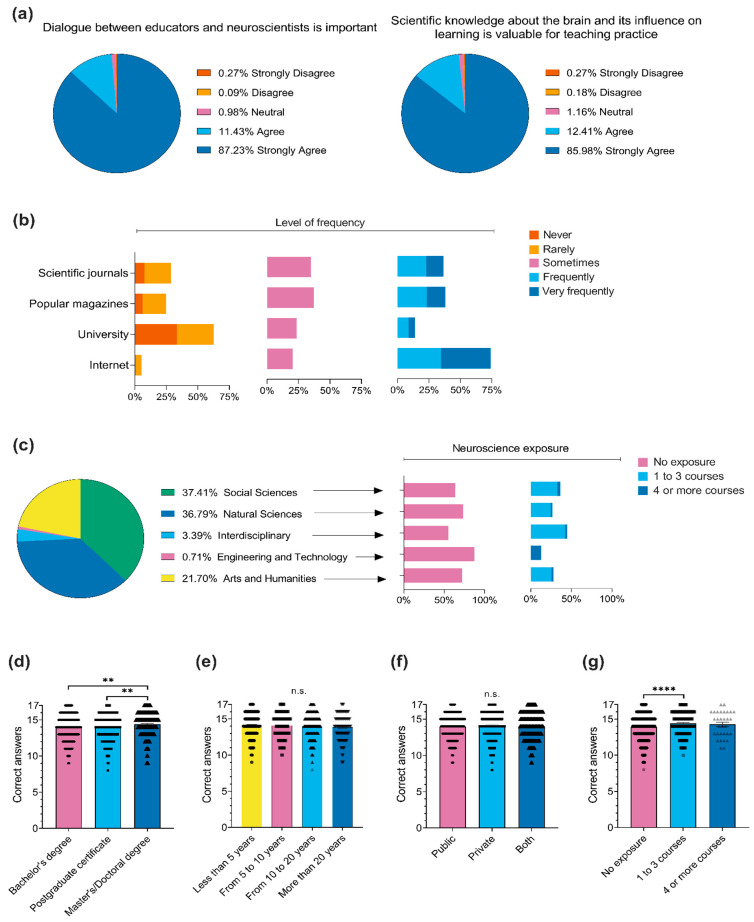
Sociodemographic characteristics, neuroscience exposure, and general neuroscience knowledge of Brazilian basic education teachers. (**a**) Participants’ ratings on the importance of dialogue between educators and neuroscientists and the relevance of neuroscience knowledge for teaching practice. (**b**) Frequency of teachers seeking scientific information from specific sources. (**c**) Distribution of participants according to fields of knowledge and corresponding levels of neuroscience exposure. Additionally, between-group comparisons of general neuroscience knowledge are presented according to (**d**) educational level, (**e**) length of teaching experience, (**f**) type of school institution, and (**g**) level of previous neuroscience exposure. Values in panels (**d**–**g**) are expressed as means ± SEM. Statistical significance is indicated as ** *p* < 0.01, **** *p* < 0.0001, and ‘n.s.’ indicates no significant differences between groups. Each black triangle denotes an individual response, illustrating data distribution within each group.

Regarding previous exposure to neuroscience extracurricular courses, 68.93% (*n* = 772) had not taken any, 28.30% (*n* = 317) had taken one to three courses, and 2.77% (*n* = 31) had taken four or more. With respect to participants’ perceptions regarding the relevance of neuroscience (Figure 1a), 98.66% (*n* = 1105) either agreed or strongly agreed that dialogue between educators and neuroscientists is important. Additionally, 98.39% (*n* = 1102) agreed or strongly agreed that scientific knowledge about the brain and its impact on learning is valuable for their teaching practices. In relation to previous exposure to scientific and neuroscientific information (Figure 1b), a substantial proportion of participants reported never or rarely seeking information in scientific journals (28.57%, *n* = 320), in popular magazines (24.82%, *n* = 278), or receiving brain-related content in their workplace (e.g., lectures, workshops) (62.50%, *n* = 700). Nonetheless, the majority reported frequently or very frequently searching for scientific information on the internet (62.70%, *n* = 702).

In terms of the distribution of participants across different fields of knowledge (Figure 1c), 37.41% (*n* = 419) were in Social Sciences, 36.79% (*n* = 412) in Natural Sciences, 21.70% (*n* = 243) in Arts and Humanities, 3.39% (*n* = 38) in Interdisciplinary, and 0.71% (*n* = 8) in Engineering and Technology. Participants from the Interdisciplinary (44.74%, *n* = 17) and Social Sciences (36.52%, *n* = 153) fields reported the highest exposure to neuroscience (i.e., at least one extracurricular course).

Across the general neuroscience knowledge questionnaire, the average percentage of incorrect responses was 17.42%. The items with the highest error rates were those related to how substances enter the nervous system, the functions of brain regions, and how the brain learns (Table A2 in Appendix A). Regarding participants’ performance on the neuroscience questionnaire, there were significant differences in performance based on educational level (*F*_(2,1117)_ = 6.26, *p* = 0.002, *η*^2^ = 0.01, Figure 1d). Specifically, participants with a master’s/doctoral degree scored significantly higher than those with a postgraduate certificate (*p* = 0.003, *d* = 0.27) and a bachelor’s degree (*p* = 0.005, *d* = 0.26). However, there was consistency regardless of length of teaching experience (*F*_(3,1116)_ = 1.27, *p* = 0.34, *η*^2^ = 0.003, Figure 1e) and the type of school institution (*F*_(2,1117)_ = 1.74, *p* = 0.18, *η*^2^ = 0.002, Figure 1f). Additionally, there were significant differences in performance across neuroscience exposure levels (*F*_(2,1117)_ = 10.81, *p* < 0.001, *η*^2^ = 0.02, Figure 1g). Participants who attended one to three courses scored significantly higher than those with no exposure (*p* < 0.0001, *d* = 0.36). Overall, these data suggest that while the length of teaching experience does not significantly influence participants’ general neuroscience knowledge, differences emerge based on educational levels and varying levels of neuroscience exposure.

### 3.3. Teachers’ Self-Efficacy and Previous Neuroscience Exposure

**Figure 2:**
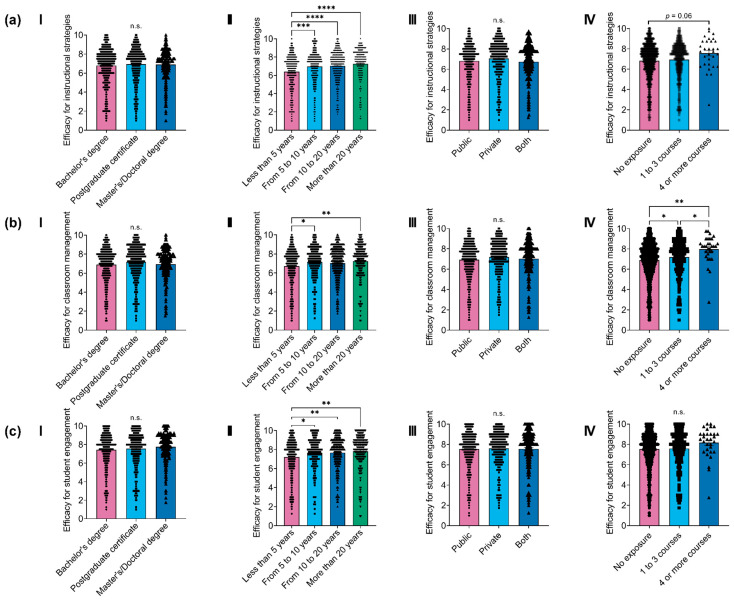
Between-group comparisons of teachers’ self-efficacy scores across three dimensions: (**a**) Efficacy for instructional strategies, (**b**) efficacy for classroom management, and (**c**) efficacy for student engagement. For each dimension, comparisons were made according to (**I**) educational level, (**II**) length of teaching experience, (**III**) type of school institution, and (**IV**) level of previous neuroscience exposure. Values are expressed as means ± SEM. Statistical significance is indicated as * *p* < 0.05, ** *p* < 0.01, *** *p* < 0.001, **** *p* < 0.0001, and ‘n.s.’ indicates no significant differences between groups. Each black triangle denotes an individual response, illustrating data distribution within each group.

The TSES scores were analyzed across three dimensions. The results showed that efficacy for instructional strategies had the highest mean (M = 7.57, SD = 1.79) with high internal consistency (Cronbach’s α = 0.94). Efficacy for student engagement was intermediate (M = 7.02, SD = 1.78), also with strong internal consistency (α = 0.91). In contrast, efficacy for classroom management had the lowest mean (M = 6.88, SD = 1.80), though it still demonstrated strong internal consistency (α = 0.93). This high reliability suggests that items within each dimension are internally consistent, supporting the scale’s validity and reliability. The results of the comparisons in the three TSES dimensions are presented in Figure 2.

Participants’ efficacy for instructional strategies did not significantly differ by educational level (*F*_(2,1117)_ = 0.61, *p* = 0.54, *η*^2^ = 0.001, Figure 2a-I) or the type of school institution (*F*_(2,1117)_ = 2.58, *p* = 0.08, *η*^2^ = 0.005, Figure 2a-III). However, it was higher in individuals with longer teaching experience (*F*_(3,1116)_ = 11.95, *p* < 0.001, *η*^2^ = 0.03, Figure 2a-II) and those with previous neuroscience exposure (*F*_(2,1117)_ = 2.96, *p* = 0.05, *η*^2^ = 0.005, Figure 2a-IV). Specifically, participants with more than 20 years of experience (*p* < 0.0001, *d* = 0.49), 10 to 20 years (*p* < 0.0001, *d* = 0.37), and 5 to 10 years (*p* < 0.001, *d* = 0.32) reported significantly higher efficacy for instructional strategies than those with fewer than 5 years. Additionally, those who had attended four or more extracurricular neuroscience courses had marginally higher scores than those with no previous neuroscience exposure (*p* = 0.06, *d* = 0.42).

Regarding participants’ efficacy for classroom management, it was also consistent across educational levels (*F*_(2,1117)_ = 2.79, *p* = 0.06, *η*^2^ = 0.005, Figure 2b-I) and types of school institutions (*F*_(2,1117)_ = 2.41, *p* = 0.09, *η*^2^ = 0.004, Figure 2b-III), but was higher among individuals with longer teaching experience (*F*_(3,1116)_ = 4.72, *p* = 0.003, *η*^2^ = 0.01, Figure 2b-II) and previous neuroscience exposure (*F*_(2,1117)_ = 7.50, *p* < 0.001, *η*^2^ = 0.01, Figure 2b-IV). Participants with more than 20 years of experience (*p* < 0.01, *d* = 0.30) and 5 to 10 years (*p* = 0.02, *d* = 0.25) reported significantly higher efficacy for classroom management than those with fewer than 5 years. Moreover, those who had attended one to three (*p* = 0.05, *d* = 0.16) and four or more (*p* < 0.01, *d* = 0.60) extracurricular neuroscience courses had significantly higher efficacy than the participants with no neuroscience exposure. Notably, participants with four or more courses also had higher scores than those with one to three (*p* = 0.05, *d* = 0.45).

With respect to participants’ efficacy for student engagement, it was consistent across the educational level (*F*_(2,1117)_ = 2.41, *p* = 0.09, *η*^2^ = 0.004, Figure 2c-I), type of school institution (*F*_(2,1117)_ = 0.14, *p* = 0.87, w + *η*^2^ = 0.0003, Figure 2c-III), and previous neuroscience exposure (*F*_(2,1117)_ = 1.91, *p* = 0.15, *η*^2^ = 0.003, Figure 2c-IV). However, it was higher in individuals with longer teaching experience (*F*_(3,1116)_ = 6.30, *p* < 0.001, *η*^2^ = 0.02, Figure 2c-II). Participants with more than 20 years of experience (*p* < 0.001, *d* = 0.34), 10 to 20 years (*p* = 0.004, *d* = 0.26), and 5 to 10 years (*p* = 0.01, *d* = 0.26) reported significantly higher efficacy for student engagement than those with fewer than 5 years.

### 3.4. Analysis of Predictors for Teachers’ Self-Efficacy

Multiple linear regression models were constructed to examine the predictors of the three dimensions of the TSES. Each model was progressively expanded by incorporating additional control variables to evaluate their impact on efficacy for instructional strategies (Table 1), classroom management (Table 2), and student engagement (Table 3). For all three dimensions, Model 1 included educational level, length of teaching experience, type of school institution, and neuroscience exposure as predictors. Model 2 introduced gender, while Model 3 added age as a control variable.

#### 3.4.1. Efficacy for Instructional Strategies

In Model 1 (Adjusted *R*^2^ = 0.03, *F*_(7,1112)_ = 6.33, *p* < 0.001), longer teaching experience emerged as a positive predictor of efficacy for instructional strategies (*B* = 0.29, *p* < 0.001). Conversely, working in a public school (*B* = −0.29, *p* = 0.02) or in both public and private institutions (*B* = −0.38, *p* = 0.02) was associated with lower self-efficacy. Additionally, higher levels of neuroscience exposure (i.e., four or more extracurricular courses) also predicted increased self-efficacy (*B* = 0.71, *p* = 0.03).

In Model 2 (Adjusted *R*^2^ = 0.04, *F*_(8,1111)_ = 6.05, *p* < 0.001), gender (i.e., men) was a significant positive predictor (*B* = 0.24, *p* = 0.05). The overall findings remained similar: teaching experience (*B* = 0.30, *p* < 0.001) and neuroscience exposure (*B* = 0.73, *p* = 0.03) continued to be significant positive predictors of efficacy for instructional strategies, while working in a public school (*B* = −0.30, *p* = 0.02) or both public and private institutions (*B* = −0.41, *p* = 0.01) remained negative predictors.

In Model 3 (Adjusted *R*^2^ = 0.03, *F*_(9,1110)_ = 5.37, *p* < 0.001), age did not significantly predict efficacy for instructional strategies (*B* = 0.001, *p* = 0.91). The significance of the other predictors remained stable, with teaching experience (*B* = 0.29, *p* < 0.001) and neuroscience exposure (*B* = 0.73, *p* = 0.03) continuing to have a positive effect, while working in a public school (*B* = −0.30, *p* = 0.02) or both public and private institutions (*B* = −0.41, *p* = 0.01) remained negative predictors. Additionally, gender (men) retained its significance (*B* = 0.24, *p* = 0.05), reinforcing the trend observed in Model 2.

#### 3.4.2. Efficacy for Classroom Management

For efficacy in classroom management, Model 1 (Adjusted *R*^2^ = 0.02, *F*_(7,1112)_ = 4.61, *p* < 0.001) showed that the length of teaching experience was a positive predictor (*B* = 0.15, *p* < 0.01). Education level did not show significant effects, while working in public schools (*B* = −0.28, *p* = 0.02) had a negative impact on self-efficacy. The number of neuroscience courses attended was significant for participants who completed one to three (*B* = 0.24, *p* = 0.04) and four or more courses (*B* = 1.01, *p* < 0.01).

In Model 2 (Adjusted *R*^2^ = 0.02, *F*_(8,1111)_ = 4.10, *p* < 0.001), adding gender did not result in a significant effect (*B* = 0.09, *p* = 0.47). The length of teaching of experience (*B* = 0.15, *p* < 0.01), participation in one to three (*B* = 0.24, *p* = 0.04) and four or more neuroscience courses (*B* = 1.01, *p* < 0.01), and working in public schools (*B* = −0.28, *p* = 0.02) remained significant predictors.

Finally, in Model 3 (Adjusted *R*^2^ = 0.02, *F*_(9,1110)_ = 4.04, *p* < 0.001), the inclusion of age as a control variable reduced the significance of teaching experience (*B* = 0.09, *p* = 0.12), suggesting that age may account for part of the effect previously attributed to experience. However, working in public schools (*B* = −0.29, *p* = 0.02), and participation in one to three (*B* = 0.25, *p* = 0.03) and four or more neuroscience courses (*B* = 1.01, *p* < 0.01) remained significant predictors.

#### 3.4.3. Efficacy for Student Engagement

In Model 1 (Adjusted *R*^2^ = 0.01, *F*_(7,1112)_ = 3.04, *p* < 0.01), the length of experience was a significant predictor of the efficacy for student engagement (*B* = 0.18, *p* < 0.001). Educational level did not have a significant effect, and working in public (*B* = −0.09, *p* = 0.45) or both types of school institutions (*B* = −0.09, *p* = 0.57) did not influence this dimension. Additionally, completing four or more courses had only a marginally significant effect (*B* = 0.58, *p* = 0.08).

In Model 2 (Adjusted *R*^2^ = 0.01, *F*_(8,1111)_ = 2.97, *p* < 0.01), gender (men) was not significant for self-efficacy in student engagement (*B* = 0.18, *p* = 0.12). The length of experience remained a significant predictor (*B* = 0.19, *p* < 0.001), while the effect of completing four or more neuroscience courses remained marginally significant (*B* = 0.59, *p* = 0.07).

Finally, in Model 3 (Adjusted *R*^2^ = 0.01, *F*_(9,1110)_ = 2.71, *p* < 0.01), adding age did not alter the significance of teaching experience (*B* = 0.21, *p* < 0.001) or the marginal effect of greater neuroscience exposure (*B* = 0.59, *p* = 0.07). However, age itself was not a significant predictor of self-efficacy for student engagement (*B* = −0.01, *p* = 0.42).

## 4. Discussion

This study investigated whether prior exposure to neuroscience predicts teaching self-efficacy among Brazilian basic education teachers. The core objective of this research was to address three key research questions: (1) Does neuroscience knowledge differ among Brazilian basic education teachers based on the institution type, educational level, length of teaching experience, and previous neuroscience exposure? (2) Does teaching self-efficacy vary across institution types, educational levels, length of teaching experience, and previous neuroscience exposure in Brazilian basic education teachers? (3) What are the significant predictors of higher teaching self-efficacy among Brazilian basic education teachers, considering sociodemographic factors, teaching profiles, and previous neuroscience exposure? Based on these questions, it has been hypothesized that prior exposure to neuroscience (i.e., extracurricular neuroscience) is associated with higher levels of teaching self-efficacy.

Our analysis provides the following responses to the research questions: (1) Neuroscience knowledge levels did not differ significantly by institution type or teaching experience. Teachers with higher educational levels (master’s/doctoral degrees) and those with some exposure to extracurricular neuroscience courses (one to three courses) demonstrated better scores on knowledge assessments. (2) Self-efficacy did not differ significantly by institution type or educational level but was notably higher in teachers with more teaching experience across all three TSES dimensions. Moreover, teachers exposed to extracurricular neuroscience courses reported higher efficacy in classroom management, though with low effect sizes. It is important to note that for the instructional strategies dimension, the relationship with neuroscience exposure reached only marginal significance, suggesting a weaker and less consistent association that requires cautious interpretation. This differential impact across TSES dimensions may reflect the content emphasis of typical neuroscience courses, which might address cognitive processes and behavior management more directly than student motivation and engagement. (3) Teaching experience emerged as a significant predictor in all three TSES dimensions. For instructional strategies and classroom management, attendance at multiple extracurricular neuroscience courses corresponded to higher self-efficacy, whereas teaching in public institutions was consistently associated with lower self-efficacy. The relationship between neuroscience exposure and student engagement self-efficacy was less robust, with a marginally significant result. Additionally, male teachers reported slightly higher self-efficacy in instructional strategies, while age did not significantly affect any dimension. These results underscore the potential influence of professional experience, neuroscience education, and institutional context on teachers’ confidence and expand our previous data among undergraduate students, which showed that previous exposure to neuroscience was also associated with higher levels of self-efficacy (Ribeiro et al., 2025). Aside from the small proportion of variance explained by our models, previous neuroscience exposure was a significant predictor of enhanced self-efficacy in this group. Given that, the complex interplay of these factors suggests that enhancing teacher self-efficacy likely requires multifaceted approaches, which, in our discussion, will focus on the contributions of neuroscience education to enhancing teaching self-efficacy.

### 4.1. Previous Neuroscience Exposure

In our sample, nearly 70% of participants reported no prior exposure to extracurricular neuroscience courses. This finding suggests that, despite the availability of such courses in Brazil (Lima et al., 2020), many Brazilian primary school teachers have not pursued them, possibly due to limited access, institutional constraints, or the absence of supportive public policies (Simões et al., 2022). Nonetheless, almost the entire sample recognized the importance of dialogue between educators and neuroscientists and valued scientific knowledge about the brain and its influence on learning. This discrepancy between teachers’ positive perceptions of neuroscience and their low enrollment in relevant courses signals a clear need for increased training opportunities.

Another notable outcome is that teachers with higher educational levels (master’s/doctoral degrees) and previous neuroscience course exposure (one to three) scored better on the general neuroscience knowledge questionnaire, mirroring results from prior research (Macdonald et al., 2017). However, teachers who completed four or more neuroscience courses did not show correspondingly higher knowledge levels, which may reflect diminishing returns of additional courses, potential quality variations in course content, or the possibility that highly motivated teachers pursue multiple courses regardless of their baseline knowledge retention. This aligns with the view that advanced academic training and direct engagement with neuroscience topics are critical for acquiring and retaining accurate knowledge. Consequently, incorporating neuroscience into both initial and continuing teacher training programs is vital, especially in Brazil, where educators’ neuroscience awareness remains limited (Simões et al., 2022). Additionally, the absence of differences by institution type raises questions about whether institutional resources and support structures, which often vary between public and private settings, play a less significant role in knowledge acquisition than individual professional development choices.

Together, these findings reinforce the urgency of improving communication between neuroscientists and educators. Inaccurate beliefs about the brain, known as neuromyths, remain widespread and can negatively affect teaching practices (Herculano-Houzel, 2002; Simões et al., 2022). Previous studies (Abdelkrim et al., 2020; Cuevas et al., 2023) have shown that educators often hold misconceptions about how the brain functions. While debunking these myths is important, a broader emphasis on accurate neuroscience knowledge can be more effective.

Of particular note is the high percentage of participants who reported frequently seeking scientific and neuroscientific information on the internet. This behavior may reflect both the accessibility of digital resources and a genuine interest in integrating scientific knowledge into teaching. However, it also raises concerns about the reliability and quality of the information being accessed, especially in the absence of formal training. These findings align with previous research involving undergraduate students, which similarly identified the internet as a primary source of neuroscience-related information (Ribeiro et al., 2025). In this sense, well-structured and accessible neuroscience education initiatives are essential to promote evidence-based teaching and bridge the gap between neuroscience and educational practice.

### 4.2. Teacher Self-Efficacy

Our results indicate that teacher self-efficacy was significantly influenced by professional experience and exposure to extracurricular neuroscience courses. Teachers with longer careers reported higher levels of self-efficacy in all three dimensions of the TSES: student engagement, instructional strategies, and classroom management. This finding is consistent with Bandura’s (Bandura, 1997, 2018) emphasis on direct experience as a primary source of self-efficacy. These direct experiences, accumulated through years of classroom practice, likely allow teachers to build their confidence, refine their strategies, and respond more effectively to challenges. Notably, having a higher educational level (e.g., a postgraduate certificate or master’s/doctoral degree) did not yield significantly different self-efficacy scores compared with lower educational levels, possibly because advanced degrees do not necessarily translate into more direct teaching experience (Emmers et al., 2019). While academic training can broaden theoretical knowledge and critical reflection, it may lack the situated and practical engagement required to build confidence in classroom settings.

Moreover, exposure to neuroscience courses significantly predicted teacher self-efficacy, particularly for classroom management and instructional strategies. Exposure to neuroscience courses did not significantly predict teacher self-efficacy in the student engagement dimension, suggesting that previous exposure to neuroscience may be less connected to the relational and emotional competencies required to actively engage students. These findings resonate with previous studies (Brick et al., 2021; Dubinsky et al., 2013, 2019; MacNabb et al., 2006), suggesting that familiarity with neuroscientific concepts fosters teachers’ motivation and a sense of responsibility for student outcomes. The impact of neuroscience exposure was especially pronounced among more experienced teachers, who may be better equipped to integrate new knowledge into their existing pedagogical framework. Less experienced teachers, however, may struggle to apply neuroscientific concepts without adequate institutional support, underscoring the need for well-structured policies and professional development programs, particularly relevant in Brazil, where neuroscience literacy among educators remains limited (Simões et al., 2022).

Another relevant finding is that teachers in public institutions reported lower self-efficacy in instructional strategies and classroom management compared to their counterparts in private institutions. These results reflect the structural and resource disparities between public and private schools in Brazil, where public institutions often face challenges such as inadequate infrastructure, excessive workloads, and administrative constraints (Ferreira, 2017; Sampaio & Guimarães, 2009). Such factors may undermine teachers’ confidence in their ability to implement effective pedagogical practices, regardless of their training or exposure to neuroscience. In this sense, lower self-efficacy scores should not be interpreted as a reflection of reduced ability, but rather as indicators of an environment that does not sufficiently support professional autonomy and pedagogical practice, therefore creating challenging work conditions regarding teacher motivation and perceived effectiveness, particularly in public institutions (OECD, 2021).

Finally, male teachers reported slightly higher self-efficacy in instructional strategies than female teachers, mirroring research on gender differences in teacher self-efficacy (Abdel-Hadi, 2020; Sak, 2015). The gender differences in teaching self-efficacy observed in this study may be explained by cultural and educational factors that influence perceptions of competence, particularly in the domains associated with science and technical knowledge. Prior research has shown that in science education, male teachers tend to report greater teaching self-efficacy, which may stem from longstanding gender stereotypes that position men as more capable or confident in scientific domains (Kiviet & Mji, 2003). This pattern appears to extend to neuroscience-related content, possibly reflecting similar sociocultural dynamics. However, factors such as heavier workloads and elevated job stress may also negatively impact self-efficacy beliefs (Klassen & Chiu, 2010). In that sense, lower self-efficacy among women may not reflect reduced competence or interest, but rather the influence of systemic elements that undermine confidence and perceived teaching effectiveness. These results should therefore not be seen as essentialist reflections of gender-based abilities, but rather as indicators of underlying disparities in educational experiences and workplace conditions. These findings underscore the need for professional development programs that provide equitable support to both male and female educators, fostering environments where all teachers can strengthen their self-efficacy through accessible, inclusive, and context-sensitive learning opportunities.

Taken together, the findings of this study reveal a complex interplay between prior neuroscience exposure, professional experience, and institutional context in shaping Brazilian teachers’ self-efficacy. While the data support the hypothesis that neuroscience education can contribute positively to teachers’ confidence, particularly in domains such as classroom management and instructional strategies, this association is nuanced and mediated by broader structural and demographic factors. The limited neuroscience exposure among the majority of participants, despite their favorable views toward the relevance of brain-based knowledge, highlights a persistent gap between intention and opportunity. Furthermore, disparities in self-efficacy by gender and institution type point to deeper inequalities in working conditions, access to training, and sociocultural expectations that must be addressed through targeted educational policies. As such, the promotion of neuroscience education alone is not sufficient. It must be embedded within comprehensive, equitable, and context-aware professional development strategies.

### 4.3. Strengths and Limitations

A major strength of this study is its large sample (*n* = 1120) of Brazilian basic education teachers, enhancing the representativeness of our findings and permitting nuanced analyses across various institutional contexts, educational levels, and demographic profiles. Our use of the well-validated TSES further bolsters confidence in the reliability of our measures. Additionally, the combination of descriptive analyses, group comparisons, and multiple linear regression models yielded a comprehensive investigation of the predictors of teacher self-efficacy, building on earlier work that emphasizes self-efficacy as a mediator of professional performance.

Despite identifying significant predictors of teaching self-efficacy, this study has some limitations. First, the low Adjusted *R*^2^ values (0.01–0.04) indicate that our models explain only a small proportion of the variance in teacher self-efficacy. This suggests that while neuroscience exposure and other factors identified in this study do influence teaching self-efficacy, their practical effect sizes are low. Such small explanatory power is not uncommon in educational and psychological research, where human behavior and perceptions are influenced by numerous interacting variables, many of which may be difficult to measure or were not included in our models. The limited explained variance underscores the multifaceted nature of teacher self-efficacy, which likely depends on a constellation of additional factors beyond those captured in our study, such as institutional support, classroom experience, and workload management (Duan et al., 2024; Zee & Koomen, 2016).

Second, our sampling methodology presents limitations for generalizability. The use of snowball sampling introduces inherent selection bias, potentially overrepresenting participants with existing interest in neuroscience or education-related topics. This approach typically lacks representativeness, as it relies on referral chains within social networks that often share similar characteristics and viewpoints. Additionally, self-selection bias may have skewed results toward individuals with stronger opinions about neuroscience or higher baseline self-efficacy levels. Also, the sociodemographic profile of our sample further constrains generalizability. Our data show significant regional concentration in Southeast Brazil, gender imbalance, and ethnic disproportion. These sociodemographic imbalances, combined with the methodological limitations of snowball sampling, further restrict the generalizability of our findings to the broader population of Brazilian teachers.

Third, this study relied exclusively on self-reported data for both previous neuroscience exposure and teacher self-efficacy, which may be subject to social desirability or recall biases. Self-reported measures of teacher self-efficacy may be susceptible to social desirability bias, as teachers might overestimate their capabilities to align with professional expectations or social norms within educational settings. Similarly, retrospective reporting of neuroscience exposure is vulnerable to recall biases, with participants potentially misremembering the timing, duration, or content of their neuroscience education experiences. Additionally, different neuroscience exposure experiences—ranging from brief seminars to intensive postgraduate programs—were treated equivalently in our categorization, which could have diluted the associations observed.

Fourth, the cross-sectional design precludes definitive causal inferences regarding whether neuroscience exposure directly enhances self-efficacy or whether teachers with higher self-efficacy are more inclined to seek neuroscience-related knowledge. Fifth, professional performance was not directly assessed, although it is a crucial outcome potentially influenced by self-efficacy. In the Brazilian context, variations in evaluation methods across different teaching institutions and educational levels pose additional challenges to such assessments. Finally, our sample was limited to Brazilian middle and high school teachers, which may affect the generalizability of the results to other cultural, institutional, or educational contexts.

Nonetheless, this study offers important insights into the role of continued education in neuroscience as a means to inform targeted interventions that enhance teaching confidence. By investigating whether prior neuroscience exposure can predict teaching self-efficacy, it addresses a relevant and underexplored intersection between cognitive science and educational practice. In doing so, it contributes to the growing body of research that seeks to understand how scientific knowledge about learning processes can empower educators in their daily practice. However, neuroscience education alone is unlikely to substantially transform teachers’ professional self-efficacy; rather, it should be viewed as one element within a broader ecosystem of professional development. Therefore, while promoting neuroscience education is valuable, its implementation must be integrated with policies that foster comprehensive, inclusive, and context-sensitive support systems for teachers. This reinforces the importance of the present study, which not only identifies an influential factor in self-efficacy development but also calls attention to the multifaceted nature of teacher growth in complex educational settings like those found in Brazil.

### 4.4. Implications for Practice

These findings have significant implications for educational practice, particularly regarding teacher training. Integrating neuroscience into both initial and continuing education programs appears to deepen teachers’ knowledge and enhance their confidence and competence in the classroom. Such programs can tackle common neuromyths and offer practical strategies rooted in evidence-based neuroscience. They can also be tailored to meet the specific needs of different educational contexts, including public and private schools, where resource gaps may influence teacher self-efficacy. Equally important is the need for public policies that promote equitable access to neuroscience-oriented professional development, particularly in public institutions where teachers reported lower self-efficacy. Providing infrastructure, mentorship, and institutional support can help mitigate the disparities observed in this study.

Importantly, while the effect sizes observed were low, this does not diminish the relevance of neuroscience exposure as a significant predictor. Rather, it suggests that neuroscience education alone is unlikely to improve teachers’ self-efficacy substantially and should be considered as one component within a broader, multifaceted approach to professional development. Future research should further examine the effects of specific neuroscience training programs through longitudinal designs, investigating both teacher self-efficacy and student learning outcomes. It would also be valuable to explore how different training formats influence teachers’ retention and application of neuroscientific knowledge. To enhance the representativeness of the findings, future research would also benefit from more robust sampling strategies, capable of capturing the diversity of the Brazilian teaching population. Additionally, qualitative methods could provide deeper insights into teachers’ experiences as they integrate neuroscientific knowledge into real-world classroom settings.

## 5. Conclusions

In conclusion, this study demonstrates a significant association between prior neuroscience exposure and higher teaching self-efficacy among Brazilian basic education teachers. Both professional experience and participation in extracurricular neuroscience courses emerged as predictors of increased self-efficacy, particularly in instructional strategies and classroom management. Although advanced academic qualifications were associated with higher neuroscience knowledge, they did not necessarily predict stronger self-efficacy, suggesting that degrees alone are insufficient without opportunities for applied, reflective practice. Likewise, while exposure to neuroscience courses contributed positively to some aspects of self-efficacy, it did not significantly impact the student engagement factor, indicating that additional contextual and relational variables likely influence this domain. Altogether, these findings underscore the relevance of neuroscience education as a promising, though not standalone, strategy to enhance teaching self-efficacy. Strengthening teacher self-efficacy requires not only scientific knowledge but also structural support, equitable opportunities, and policies that value and invest in the professional growth of all teachers.

## Figures and Tables

**Table 1:** Results of linear regression models for efficacy of instructional strategies.

Variables	*B*	SE(*B*)	*t*	*p*-Value
Model 1				
Intercept	6.39	0.16	40.44	<0.001 ***
Length of teaching experience	0.29	0.05	5.65	<0.001 ***
Educational level (postgraduate certificate) ^a^	−0.03	0.12	−0.26	0.79
Educational level (master’s/doctoral degree) ^a^	−0.09	0.15	−0.62	0.54
Type of institution (public) ^b^	−0.29	0.12	−2.35	0.02 *
Type of institution (both) ^b^	−0.38	0.17	−2.27	0.02 *
Neuroscience Exposure (1 to 3 courses) ^c^	0.10	0.12	0.81	0.42
Neuroscience Exposure (4 or more courses) ^c^	0.71	0.33	2.18	0.03 *
Model 2				
Intercept	6.31	0.16	38.75	<0.001 ***
Length of teaching experience	0.30	0.05	5.76	<0.001 ***
Educational level (postgraduate certificate) ^a^	−0.02	0.12	−0.15	0.88
Educational level (master’s/doctoral degree) ^a^	−0.09	0.15	−0.60	0.55
Type of institution (public) ^b^	−0.30	0.12	−2.41	0.02 *
Type of institution (both) ^b^	−0.41	0.17	−2.46	0.01 **
Neuroscience Exposure (1 to 3 courses) ^c^	0.11	0.12	0.93	0.35
Neuroscience Exposure (4 or more courses) ^c^	0.73	0.33	2.24	0.03 *
Gender (Men) ^d^	0.24	0.12	1.98	0.05 *
Model 3				
Intercept	6.29	0.27	23.70	<0.001 ***
Length of teaching experience	0.29	0.06	4.85	<0.001 ***
Educational level (postgraduate certificate) ^a^	−0.02	0.12	−0.16	0.87
Educational level (master’s/doctoral degree) ^a^	−0.09	0.15	−0.61	0.55
Type of institution (public) ^b^	−0.30	0.12	−2.41	0.02 *
Type of institution (both) ^b^	−0.41	0.17	−2.46	0.01 **
Neuroscience Exposure (1 to 3 courses) ^c^	0.11	0.12	0.93	0.35
Neuroscience Exposure (4 or more courses) ^c^	0.73	0.33	2.24	0.03 *
Gender (Men) ^d^	0.24	0.12	1.97	0.05 *
Age	0.001	0.01	0.11	0.91

Note: Statistics: estimated (*B*), standard error (SE), t-value (*t*), and *p*-value (*p*). Significance levels are indicated as follows: *** *p* < 0.001; ** *p* < 0.01; * *p* < 0.05. Model 1: *R*^2^ = 0.04; adjusted *R*^2^: 0.03. Model 2: *R*^2^ = 0.04; adjusted *R*^2^: 0.04. Model 3: *R*^2^ = 0.04; adjusted *R*^2^: 0.03. ^a^ Reference = bachelor’s degree. ^b^ Reference = private institution. ^c^ Reference = without neuroscience exposure. ^d^ Reference = women.

**Table 2:** Results of linear regression models for efficacy for classroom management.

Variables	*B*	SE(*B*)	*t*	*p*-Value
Model 1				
Intercept	6.70	0.16	42.80	<0.001
Length of teaching experience	0.15	0.05	2.94	<0.01 **
Educational level (postgraduate certificate) ^a^	0.15	0.12	1.25	0.21
Educational level (master’s/doctoral degree) ^a^	−0.06	0.15	−0.41	0.68
Type of institution (public) ^b^	−0.28	0.12	−2.29	0.02 *
Type of institution (both) ^b^	−0.21	0.17	−1.26	0.21
Neuroscience Exposure (1 to 3 courses) ^c^	0.24	0.12	2.02	0.04 *
Neuroscience Exposure (4 or more courses) ^c^	1.01	0.32	3.11	<0.01 **
Model 2				
Intercept	6.67	0.16	41.29	<0.001 ***
Length of teaching experience	0.15	0.05	2.98	<0.01 **
Educational level (postgraduate certificate) ^a^	0.16	0.12	1.29	0.20
Educational level (master’s/doctoral degree) ^a^	−0.06	0.15	−0.40	0.69
Type of institution (public) ^b^	−0.28	0.12	−2.31	0.02 *
Type of institution (both) ^b^	−0.22	0.17	−1.33	0.18
Neuroscience Exposure (1 to 3 courses) ^c^	0.24	0.12	2.06	0.04 *
Neuroscience Exposure (4 or more courses) ^c^	1.01	0.32	3.13	<0.01 **
Gender (Men) ^d^	0.09	0.12	0.72	0.47
Model 3				
Intercept	6.28	0.26	23.91	<0.001 ***
Length of teaching experience	0.09	0.06	1.56	0.12
Educational level (postgraduate certificate) ^a^	0.14	0.12	1.19	0.24
Educational level (master’s/doctoral degree) ^a^	−0.07	0.15	−0.46	0.64
Type of institution (public) ^b^	−0.29	0.12	−2.40	0.02 *
Type of institution (both) ^b^	−0.23	0.17	−1.40	0.16
Neuroscience Exposure (1 to 3 courses) ^c^	0.25	0.12	2.12	0.03 *
Neuroscience Exposure (4 or more courses) ^c^	1.01	0.32	3.12	<0.01 **
Gender (Men) ^d^	0.08	0.12	0.69	0.49
Age	0.01	0.01	1.88	0.06

Note: Statistics: estimated (*B*), standard error (SE), t-value (*t*), and *p*-value (*p*). Significance levels are indicated as follows: *** *p* < 0.001; ** *p* < 0.01; * *p* < 0.05. Model 1: *R*^2^ = 0.03; adjusted *R*^2^: 0.02. Model 2: *R*^2^ = 0.03; adjusted *R*^2^: 0.03. Model 3: *R*^2^ = 0.03; adjusted *R*^2^: 0.02. ^a^ Reference = bachelor’s degree. ^b^ Reference = private institution. ^c^ Reference = without neuroscience exposure. ^d^ Reference = women.

**Table 3:** Results of linear regression models for efficacy of student engagement.

Variables	*B*	SE(*B*)	*t*	*p*-Value
Model 1				
Intercept	7.12	0.16	44.98	<0.001 ***
Length of teaching experience	0.18	0.05	3.55	<0.001 ***
Educational level (postgraduate certificate) ^a^	0.01	0.12	0.09	0.93
Educational level (master’s/doctoral degree) ^a^	0.20	0.15	1.34	0.18
Type of institution (public) ^b^	−0.09	0.12	−0.76	0.45
Type of institution (both) ^b^	−0.09	0.17	−0.54	0.59
Neuroscience Exposure (1 to 3 courses) ^c^	0.05	0.12	0.45	0.65
Neuroscience Exposure (4 or more courses) ^c^	0.58	0.33	1.77	0.08
Model 2				
Intercept	7.06	0.16	43.23	<0.001 ***
Length of teaching experience	0.19	0.05	3.63	<0.001 ***
Educational level (postgraduate certificate) ^a^	0.02	0.12	0.17	0.87
Educational level (master’s/doctoral degree) ^a^	0.20	0.15	1.36	0.18
Type of institution (public) ^b^	−0.10	0.12	−0.81	0.42
Type of institution (both) ^b^	−0.12	0.17	−0.69	0.49
Neuroscience Exposure (1 to 3 courses) ^c^	0.07	0.12	0.55	0.58
Neuroscience Exposure (4 or more courses) ^c^	0.59	0.33	1.81	0.07
Gender (Men) ^d^	0.19	0.12	1.56	0.12
Model 3				
Intercept	7.23	0.27	27.19	<0.001 ***
Length of teaching experience	0.21	0.06	3.52	<0.001 ***
Educational level (postgraduate certificate) ^a^	0.03	0.12	0.21	0.83
Educational level (master’s/doctoral degree) ^a^	0.21	0.15	1.38	0.17
Type of institution (public) ^b^	−0.09	0.12	−0.77	0.44
Type of institution (both) ^b^	−0.11	0.17	−0.66	0.51
Neuroscience Exposure (1 to 3 courses) ^c^	0.06	0.12	0.52	0.60
Neuroscience Exposure (4 or more courses) ^c^	0.59	0.33	1.81	0.07
Gender (Men) ^d^	0.19	0.12	1.57	0.12
Age	−0.01	0.01	−0.82	0.41

Note: Statistics: estimated (*B*), standard error (SE), t-value (*t*), and *p*-value (*p*). Significance levels are indicated as *** *p* < 0.001. Model 1: *R*^2^ = 0.02; adjusted *R*^2^: 0.01. Model 2: *R*^2^ = 0.02; adjusted *R*^2^: 0.01. Model 3: *R*^2^ = 0.02; adjusted *R*^2^: 0.01. ^a^ Reference = bachelor’s degree. ^b^ Reference = private institution. ^c^ Reference = without neuroscience exposure. ^d^ Reference = women.

## Data Availability

The original data presented in the study are openly available in the Open Science Framework (OSF) at http://www.doi.org/10.17605/OSF.IO/X9N5J, accessed on 14 May 2025.

## Appendix A

**Table A1:** Sociodemographic and teaching profile data of the sample (*n* = 1120).

Sociodemographic	*n* (%)
Gender	
Woman	811 (72.41%)
Man	309 (27.59%)
Self-identified ethnicity	
White	649 (57.95%)
Brown	313 (27.95%)
Black	116 (10.36%)
Asian	14 (1.25%)
Indigenous	3 (0.27%)
Other and prefer not to answer	25 (2.23%)
Region of residence	
South	76 (6.79%)
Southeast	848 (75.71%)
Midwest	46 (4.11%)
Northeast	116 (10.36%)
North	34 (3.04%)
Income	
Less than 1 minimum wage	34 (3.04%)
1 to 3 minimum wage	245 (21.88%)
3 to 5 minimum wage	382 (34.11%)
5 to 10 minimum wage	411 (36.70%)
More than 10 minimum wage	48 (4.29%)
**Teaching profile**	***n* (%)**
Educational level	
Bachelor’s degree	426 (38.04%)
Postgraduate certificate	469 (41.88%)
Master’s/Doctoral degree	225 (20.09%)
Length of teaching experience	
Less than 5 years	313 (27.95%)
5 to 10 years	258 (23.04%)
10 to 20 years	353 (31.52%)
More than 20 years	196 (17.50%)
Type of institution	
Public	629 (56.16%)
Private	318 (28.39%)
Both	173 (15.45%)
Grade level	
Middle school	259 (23.13%)
High school	251 (22.41%)
Both	610 (54.46%)
Fields of knowledge	
Natural Sciences	412 (36.79%)
Engineering and Technology	8 (0.71%)
Social Sciences	419 (37.41%)
Arts and Humanities	243 (21.70%)
Interdisciplinary	38 (3.39%)

**Table A2:** Percentage of accurate items on the general neuroscience knowledge questionnaire.

Remaining Survey Items (Ranked by % Incorrect)	Correct Answer	%
Vaccines, electronic chips, alcohol, and any other substance present in the blood can enter the nervous system through the bloodstream.	FALSE	50.09
The left and right hemispheres of the brain work together.	TRUE	31.79
When a brain region is damaged, other parts of the brain can take up its function.	TRUE	30.18
Learning is due to the addition of new cells to the brain.	FALSE	29.29
Normal development of the human brain involves the birth and death of brain cells.	TRUE	22.50
Academic achievement can be negatively impacted by skipping breakfast.	TRUE	21.88
Information is stored in the brain in networks of cells distributed throughout the brain.	TRUE	21.43
Extended rehearsal of some mental processes can change the structure and function of some parts of the brain.	TRUE	20.54
Vigorous exercise can improve mental function.	TRUE	16.34
Brain development has finished by the time children reach puberty.	FALSE	13.93
Circadian rhythms (“body-clock”) shift during adolescence, causing students to be tired during the first lessons of the school day.	TRUE	13.75
Production of new connections in the brain can continue into old age.	TRUE	7.41
Learning occurs through changes to the connections between brain cells.	TRUE	7.23
We use our brains 24 h a day.	TRUE	4.73
Mental capacity is genetic and cannot be changed by the environment or experience.	FALSE	2.68
There are specific periods in childhood when it’s easier to learn certain things.	TRUE	2.14
When we sleep, the brain shuts down.	FALSE	0.45
Average percentage incorrect answers		17.42
